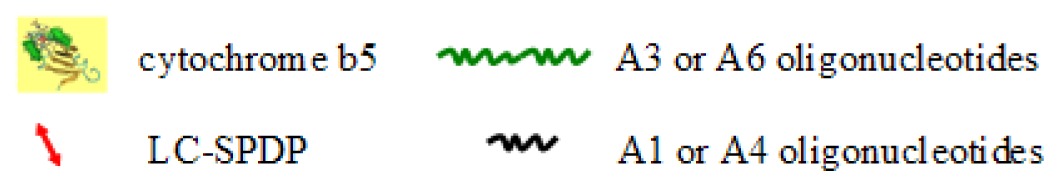# Nanobioengineering and Characterization of a Novel Estrogen Receptor Biosensor

**DOI:** 10.3390/s8074413

**Published:** 2008-07-28

**Authors:** Alexandre Berthier, Céline Elie-Caille, Eric Lesniewska, Régis Delage-Mourroux, Wilfrid Boireau

**Affiliations:** 1 Institut FEMTO-ST, Université de Franche Comté, Clinical-Innovation Proteomic Platform, CNRS – 25044 Besançon cedex, France; E-Mails: alexandre.berthier@clipproteomic.fr (Alexandre Berthier); celine.caille@clipproteomic.fr (Céline Elie-Caille); 2 Estrogènes, Expression Génique et Pathologies du Système Nerveux Central, EA3922, IFR 133, Université de Franche-Comté, UFR Sciences et Techniques, 16 route de Gray, 25030 Besançon cedex, France; E-mail: regis.delagemourroux@clipproteomic.fr (Régis Delage-Mourroux); 3 Institut Carnot Bourgogne UMR CNRS 5209, Nanosciences Department, University of Bourgogne, B.P. 47870, 21078 Dijon Cedex, France; E-mail: eric.lesniewska@clipproteomic.fr (Eric Lesniewska)

**Keywords:** Nano-objects, molecular lego, AFM, SPR, Protein/DNA interaction

## Abstract

We constructed an original supramolecular assembly on a surface of sensor composed of an innovative combination of an engineered cytochrome b5 and a modified nucleic acid bound to a synthetic lipid hemimembrane. The protein/DNA block, called (P-DNA)_2_, was synthesized and purified before its immobilization onto a hybrid bilayer reconstituted on a gold surface. Surface plasmon resonance (SPR) and atomic force microscopy (AFM) were engaged in parallel on the same substrates in order to better understand dynamic events that occur at the surface of the biosensor. Good correlations were obtained in terms of specificity and reversibility. These findings allow us to present a first application of such biosensor in the study of the interaction processes between nuclear receptor and DNA.

## 1. Introduction

Reconstitution of lipid membranes onto inorganic or metallic substrates in a biochip approach has been extensively studied during the last decade [1;2]. Especially in biosensor development, investigations on bio-molecular recognitions, interactions or captures in a biomimetic environment can be an advantage in comparison with others functionalization processes. This has been recently pointed out in many biochip approaches involving peptide or protein chips [3-5]. Moreover, a critical step in the making of these biochips was the development of appropriate surface immobilization protocols. In order to prevent steric hindrances and random organizations, the immobilization of ligands must be highly controlled. This major drawback was often occulted leading to not completely optimized sensors. Beyond the biomimetic point of view, lipid membranes have shown strong versatility in their composition leading to many lipid-based functionalization strategies [6;7]. The most common approach is based on the introduction of small fractions of modified lipids allowing chemical or biochemical immobilizations on surface. A very promising strategy, inspired by ion metallic affinity chromatography (IMAC), was developed by the Tampé and Arnold groups in 1997/1998 [8;9] and significantly developed since 2002 [10-12]. It consists of incorporating synthetic lipids bearing a nitrilotriacetic acid (NTA) or iminodiacetic acid (IDA) moieties that complex metallic divalent ions such as Cu^2+^, Zn^2+^ or Ni^2+^. Such surfaces efficiently capture histidine-tagged proteins by coordination bonds but, contrary to covalent coupling, these bonds were characterized by reversibility properties. Thus, the regeneration was obtained completely by adding metal ion chelating agents such as EDTA or competitor compounds as free histidine or imidazole [13].

We have previously immobilized on hybrid bilayers (HB) a unique supramolecular assembly of a redox protein with nucleic acids, called P-DNA blocks. The stability of this assembly was strengthened by reconstituting the complex below the transition phase of the lipid matrix. In this configuration, we have demonstrated that the P-DNA design offers the possibility to accurately control the density of immobilized probes on the bio-mimetic layer and optimize the DNA chip sensitivity and specificity [10;14]. These results have open the way for the development of a new generation of biosensors that allow analysis of the modulation of DNA–DNA and DNA–RNA interactions by a large range of chemicals or biological effectors.

In this paper, we present an original structure design based on assembly of P-DNA blocks driven by specific DNA hybridization process. The resulting supramolecular assembly is called (P-DNA)_2_. (P-DNA)_2_ blocks have been conceived to reconstitute a palindromic DNA response element called Estrogen Response Element (ERE) which is recognized by the estrogen receptor (ER), a member of the nuclear receptor super-family [15]. (P-DNA)_2_ blocks were extensively characterized by gel electrophoresis and spectrophotometric measurements. Mechanisms of reconstitution of (P-DNA)_2_ complexes onto lipid matrices were intensely investigated by combining surface plasmon resonance (SPR) and atomic force microscopy (AFM). From our knowledge, this is the first study presenting in parallel a global analysis and nanoscale characterizations of bio-molecular building blocks on exactly the same gold substrate (commercial gold chip). These complementary investigations allowed to establish a biosensor devoted to the study of DNA-protein interactions which are illustrated herein in the case of estrogen receptors.

## 2. Results

### 2.1. P-DNA supramolecular buildings

Constructions of molecular structures presenting DNA were performed in three steps:

(i) cross reaction between ssDNA and Succinimidyl 6-[3′-(2-PyridylDithio)-Propionamido] hexanoate (LC-SPDP), (ii) coupling of cytochrome b5 with LC-SPDP-ssDNA entities to form P-DNA blocks and (iii) dimerization of P-DNA through hybridization to form (P-DNA)_2_ blocks. The first two steps of synthesis that lead to P-DNA blocks were previously established [14]. Briefly, efficiency of hetero-bifunctional linker/DNA coupling was evaluated by spectrophotometric measurements and analyzed in the presence of excess DTT. In our study, the efficiency of A1/LC-SPDP coupling was 80% and A4/LC-SPDP was 75%. Then, these modified oligonucleotides were incubated with the genetic engineered cytochrome b5. A unique and highly specific protein / linker coupling was obtained due to the cystein at position 24. Unreactive compounds were eliminated by a combination of chromatographic steps (see materials and methods part) leading to highly purified P-DNA blocks. All the steps of synthesis were characterized by spectrophotometric measurements. We have determined optimal conditions to generate (P-DNA)_2_ blocks, various molecular ratios of P-DNA and overlapped complementary oligonucleotides have been tested (see supplementary result 1).

After hybridization and gel filtration processes, the composition of b5-DNA populations was determined by analysis of absorbance ratios (A_260_/A_412_) (Table 1).

Excess of P-DNA blocks corresponded to optimal conditions to synthesize (P-DNA)_2_ block majority (see supplementary result 1).

### 2.2. Building of the lipidic chip

#### 2.2.1. SPR characterization

**Figure 1.**
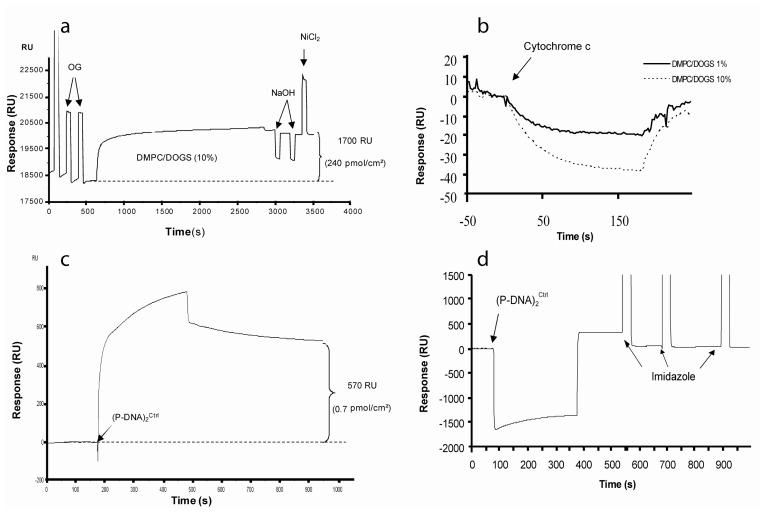
**Specific assembling of (P-DNA)_2_onto the chip. (a)** Sensorgram of the hybrid bilayer establishment. This HB was realized at 25°C, on functionalized gold surface in PB buffer. SAM was first wetted with 50% ethanol, and then washed with two pulses (1 min each) of 40 mM OG at 50 μl/min. 1 mM SUV (DOGS/DMPC 10% mol/mol) was injected at 2 μl/min and spread onto the cleaned surface. At the end of the injection, flow was increased to 20 μl/min and two pulses of 20 mM NaOH treated the lipidic surface in order to remove excess vesicles. DOGS were reloaded in Ni^2+^ by an injection of NiCl_2_ (20 mM in acetate buffer). At the end of this process, the response signal was ∼1700 RU corresponding to 240 pmol/cm^2^, **(b)** The sensorgram shows the control of the homogeneity of the HB through injections at 20 μl/min of a dummy protein (cytochrome C 1 μM). **(c)** Sensorgram of the (P-DNA)_2_^Ctrl^ anchorage. This step was realized in PBS running buffer at 25°C at a concentration of 2 μM during 5 minutes at 5 μl/min. **(d)** Sensorgram showing the specific anchorage of the (P-DNA)_2_ block. The lipidic surface was regenerated by three pulses of 0.5 M imidazole (30 seconds at 20 μl/min).

The step-by-step construction of the biochip was followed by SPR. The hydrophobic monolayer (OM) was wetted by a pulse of ethanol (50%) and washed with a non-ionic detergent, Octyl-Glucopyranoside (OG). These steps allowed cleaning the surface before the fusion of the Small Unilamellar Vesicles (SUVs). SUV were afterwards injected and, during the interaction with the surface, they spread spontaneously until reaching a plateau after 1200s. Injection was continued in order to completely form a lipidic monolayer. At the end of the fusion of SUVs onto the Self-Assembled Monolayer (SAM), two pulses of sodium hydroxide (20 mM) were used to remove lipid excess and to establish a stable dense layer. At the end of the process, the surface density of DMPC/DOGS 10% was 270 ± 0.40 pmol/cm^2^ (Figure 1a).

It is important to keep in mind that this entire process can be affected by some parameters (roughness of the substrate, atmospheric pollutions…) which can imply heterogeneity of the lipidic up-layer. For example, some holes could permit adsorption of molecules directly on the bare gold surface or on Octadecyl Mercaptan (OM) monolayer. SPR studies of the spreading and fusion are not sufficient to guarantee the integrity of the HB. We automatically performed controls by using a dummy protein that mimics the potential non-specific adsorption of the cytochrome b5 with the biochip. The main binding force was the hydrophobicity generated by the SAM down layer. The secondary forces were electrostatic interactions that can be attenuated by using a buffer with a high ionic strength. In our approach, we chose cytochrome c because of its similar conformation with cytochrome b5 (length of amino acid primary sequence, globular structure, porphyrin moiety…). Thus our procedure contains an internal marker which prevents the using of defective chips. For more than 80% of tested chips, the cytochrome c binding signals did not exceed the acceptable baseline drifts (1 RU/min) (Figure 1b).

Purified (P-DNA)_2_^Ctrl^ has been injected and quantified after assembling on the lipidic layer (Figure 1c). The mechanism of interaction of (P-DNA)_2_ with HB was determined by fitting association/dissociation results with kinetic models. Optimal fitting was obtained using the “Bivalent analyte” model (*BiaEvaluation* 3.05 software). (P-DNA)_2_ blocks bind sequentially with two lipidic anchors conferring high stability of the DNA probes (see supplementary result 2).

When (P-DNA)_2_^Ctrl^ was added at the μM range, the saturation of anchorage was usually achieved 30 min after injection yielding a maximum grafting of 800 RU, i.e closed to 10 fmole/mm^2^ (data not shown).

In order to confirm the specificity of (P-DNA)_2_^Ctrl^ anchorage, 10 μL (20 μL/min) of 0.5 M imidazole solution, a specific histidine competitor, were injected and produced a quasi complete regeneration step (i.e. more than 90% of (P-DNA)_2_ released) (Figure 1d).

#### 2.2.2. AFM characterization of (P-DNA)_2_ complexes immobilized onto the supported HB

To be relevant when compared to the SPR characterization, we chose to process AFM imaging on the same commercial gold chips used for SPR experiments.

Indeed, visualizing how supramolecular complexes behave onto the SPR-used gold supported membrane could help understanding interactions occurring closed to the surface. First, the surface of the hydrophobic gold chip was imaged, and revealed a rough surface presenting globular gold particles of around 30 nm in diameter. Second, the establishment of the supported hybrid membrane (before and after sodium hydroxide cleaning) has been investigated on small and large areas (see supplementary result 3). From the homogeneous lipidic surface, we studied the potential of (P-DNA)_2_ to bind specifically through histidine/nickel interactions.

**Figure 2.**
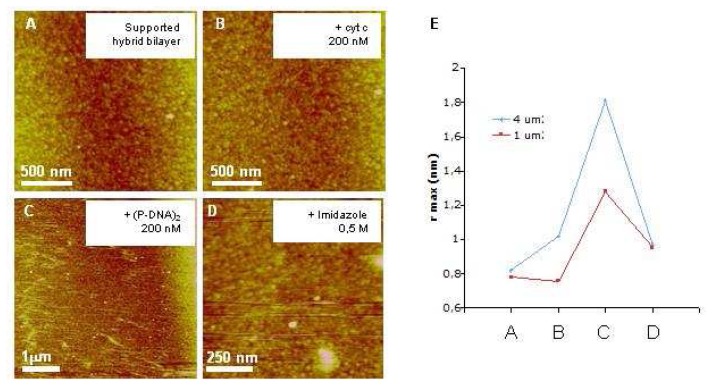
**Specificity and reversibility of the biosensor**. AFM images of gold supported HB after DOGS reloading in Ni^2+^**(a)** after 20 min incubation of 200 nM cytochrome c **(b)**, (P-DNA)_2_**(c)** and after imidazole incubation **(d)** obtained by contact mode imaging in liquid conditions. **(e)** Surface roughness (in nm) was determined on every image, on 1 and 4 mm^2^. z range corresponds to 15 nm in contact mode.

At this step, it should be mentioned that high resolution imaging of molecular assemblies on rough surface represents a challenge. Indeed, the flatter the surface the higher resolution of images obtainable. Nevertheless, it was of particular interest to visualize molecular interactions that occurs on the same surfaces employed for SPR experiments in order to connect these investigations to a realistic approach for biosensors. Thus, after (P-DNA)_2_ binding to the supported hybrid membrane, the surface modification and the grafting specificity were analyzed. While incubation with cytochrome c (200 nM) had no effect on the surface appearance (Figure 2b, that is confirmed by roughness measurements Figure 2e), (P-DNA)_2_ presentation to the surface induced a surface modification (Figure 2c), and an increase of the roughness (Figure 2e). Images obtained in contact mode showed a surface change with a slight dragging of motifs over the surface (Figure 2c). Washing the surface with 0.5 M imidazole (Figure 2d) allowed to regenerate with good efficiency the surface. The surface roughness calculation, at these different steps confirmed these observations (Figure 2e) proving both specificity and reversibility of (P-DNA)_2_ complex binding.

**Figure 3.**
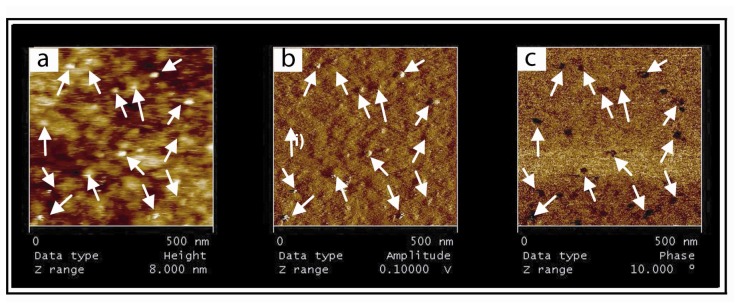
**Visualization of (P-DNA)_2_ complexes immobilized onto the supported HB.** The height **(a)** amplitude **(b)** and phase **(c)** representations from oscillating contact mode AFM images are nicely correlated after (P-DNA)_2_ incubation on the HB. White arrows indicate motifs. The supported membrane contains 1% nickel modified lipids and was reloaded with a 50 mM nickel solution in acetate buffer. The (P-DNA)_2_ complexes were incubated for 30 min onto the membrane, followed by extensive washes with PBS buffer.

After reloading DOGS with 50 mM nickel solution and a 20 min incubation with (P-DNA)_2_, oscillating contact mode AFM images at higher resolution showed globular motifs with a size corresponding to the size of a (P-DNA)_2_ complex (length ∼18nm) (Figure 3a). Amplitude and phase representations were also recorded in order to discriminate (P-DNA)_2_ more clearly from the surface (Figure 3b, c). A nice correlation between height, amplitude and phase signals confirmed the binding of (P-DNA)_2_ complexes onto the surface.

These nicely correlated results show that experiments at “molecular scale” (on 500×500 nm, thus 2.5.10^-7^ mm^2^ by AFM) represents a fine approach relating specificity and reversibility of molecular interactions. Thus, SPR and AFM results, while investigating the same surface but at different scales (1.4 mm^2^ and 2.5.10^-7^ mm^2^ respectively) are coherent.

#### 2.2.3. Paradigm of estrogen receptor sensor

**Figure 4.**
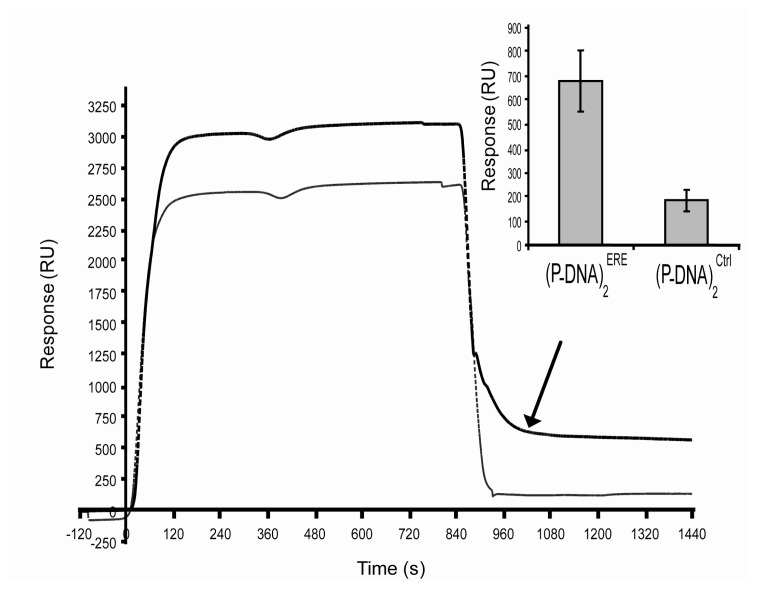
**Specific interaction between ERα and ERE target sequence.** After 4 hours incubation of 50 nM ERα with 1 nM E_2_ in PBS at 4°C, 300 μl of “activated” receptors were injected at 20 μl/min on (P-DNA)_2_^ERE^ (thick curve) or on (P-DNA)_2_^Ctrl^ (thin curve). The graphic representation of the results was the mean of four experiments.

Following the previous procedures, two populations of purified (P-DNA)_2_ blocks can be grafted on lipidic chips leading to the establishment of an estrogen receptor biosensor including ERE and reference channels in Biacore 2000 apparatus.

(P-DNA)_2_^Ctrl^ and (P-DNA)_2_^ERE^ were immobilized on the lipid matrix reconstituted on functionalized gold sensor chips leading to a homogeneous loading of 150 RU for each species. Activated estrogen receptor (50 nM ERα with 1 nM E_2_) was injected and molecular interactions followed in real time (Figure 4).

The level of association was highest on the ERE channel and dissociation event occurred on this channel whereas none was observed on the reference (control sequence) indicating a dynamic equilibrium between DNA target and protein probes. Finally at the end of ERα injections, mean values were more than 3.6 fold higher on ERE target than on control sequence (680 RU +/- 127 (10,2 fmol/mm^2^) versus 186 RU +/- 45 (2,8 fmol/mm^2^) respectively). The biosensor can be regenerated at the hybrid layer level by a clearing process of ER/(P-DNA)_2_ with two pulses of 0.5 M imidazole (data not shown).

## 3. Discussion

In the present study, a population of engineered protein derived from human microsomal cytochrome b5 specifically linked to an oligonucleotide with a hetero-bifunctional linker was synthesized to yield a building block called P-DNA. P-DNA was a versatile molecular block able to promote the formation of taller and highly controlled nano-objects. Many strategies can be used to obtain such supramolecular structures including (i) hybridization in solution followed by chromatographic steps or (ii) solid-phase synthesis strategy. Recently, the latter option was successfully used to construct large P-DNA blocks in a DNA network project [16]. In our study, reconstitution in solution was convenient in regard to the final molecular assembly. Thus, P-DNA blocks were incubated in a optimized procedure with long overlapping complementary oligonucleotides to give an unique supramolecular block called (P-DNA)_2_. At the end of hybridization process, excess P-DNA was easily separated by gel exclusion chromatography and complexes of interest were detected by non-denaturing electrophoresis. Specific stains revealed nucleic and protein parts of these blocks (see supplementary result 1). Each (P-DNA)_2_ block presented two tags with the –NGHHH-COOH sequence which allow the interaction with two lipid anchors (DOGS) through coordinated histidine/nickel binding. Thermodynamically, this structure is more strongly associated with the lipidic matrix than P-DNA and the mechanism of association/dissociation fits well with a kinetic model of “bivalent analyte” as shown in SPR experiments. Moreover, Stenberg's calibration, in SPR experiments, demonstrated that 1000 RU corresponds to 1 ng/mm^2^ of protein [17]. The amount of complexes covering the lipidic membrane as determined from SPR results was estimated taking into account molecular mass and area of 75 kDa and 100 nm^2^ respectively for the (P-DNA)_2_ blocks. Based on these theoretical dimensions, the maximum (P-DNA)_2_ coverage achieved was 16.6 fmoles/mm^2^ or 1250 RU. The amount of 1% of DOGS lipid represented the smaller amount of DOGS leading to complete (P-DNA)_2_ immobilization. Upper limit was raised to 10% DOGS lipid content in order to magnify the amount of (P-DNA)_2_ blocks immobilized. Experimental SPR data did not exceeded 800 RU following the injection of (P-DNA)_2_ blocks, which corresponds to 10 fmoles/mm^2^. Dissociation occurs at this level of loading and stabilization of the interactions was observed below 6 fmoles/mm^2^. The phenomenon of dissociation of P-DNA has previously been demonstrated and resulted from both the rupture of the chelate-mediated link and the extraction of lipid anchors from the lipidic matrix [14]. This major drawback was overcome by using two tags per (P-DNA)_2_ block. However, (P-DNA)_2_ blocks did not bind simultaneously with two DOGS. According to the availability of free DOGS in the lipid matrix, a part of them could link only one anchor domain. These “not fully bound” blocks were probably released from the surface during experiments. On the other hands, when ER sensors were established, regeneration processes using competitors of Ni^2+^ coupling or Ni^2+^ chelating agents were efficient as shown both in SPR and AFM investigations.

In order to better understand events that occur at the surface of the biochip, we performed AFM investigations of substrate employed for SPR experiments. The relevancy and originality of the present imaging study lies on the fact that visualization of specifically immobilized (P-DNA)_2_ complexes was performed on the same commercial gold chips used for SPR characterizations. While this substrate presents a rough surface, far rougher than the flat mica usually used for AFM imaging, we managed to visualize (P-DNA)_2_ complexes immobilized on a hybrid membrane and to demonstrate the specificity and reversibility of this grafting. SPR and AFM results support the model of (P-DNA)_2_ complexes immobilization through histidine/nickel interactions. Indeed, in both methods, grafting of (P-DNA)_2_ blocks is: i) DOGS membrane content dependent, ii) surface specific and iii) reversible. The combination of both techniques is still a challenge. Indeed, very few studies argue for the use of the AFM tool to visualize the organization of proteins on the same working surface. Indeed, in literature we can find AFM characterization study of sensors based on quartz crystal [12] or gold electrode [18]. But, in the first case [12], AFM experiments were actually performed on atomically flat mica, which is a deeply different surface (flat mica versus rough gold substrates). In the other study [18], while gold electrode presents a clear roughness (islands of 300 nm [19]), their AFM observations were performed on atomically flat gold (Au (111)-(1×1)). Thus, it appears not evident at all to visualize by AFM molecules and their interactions “strictly on the sensor surface”. This increases the weight of our study, since we revealed (P-DNA)_2_ complexes specifically and reversibly immobilized on similar lipid surface reconstituted on gold chip through both SPR and AFM methods.

Another challenge concerns the imaging of macromolecular complexes linked to a lipidic carriers in a lipid bilayer [20]. The lateral mobility of chelating lipids allows (P-DNA)_2_ complexes to move in the plane of membrane. Such a behavior makes high resolution imaging of complexes more difficult. Our AFM images, while highly demonstrative, are probably limited in terms of resolution due to this diffusion. At this stage, it is important to note that our biosensor offers through its structure (hybrid bilayer) the possibility to overcome this drawback. Decreasing the working temperature would indeed rigidify the supported membrane (phase transition between liquid and gel states still exist with few % of DOGS in DMPC hybrid bilayers as previously demonstrated [14]), thus limiting mobility of (P-DNA)_2_ blocks).

In order to realize an estrogen receptor biosensor, we have built two populations of (P-DNA)_2_ blocks presenting respectively the specific palindromic DNA estrogen response element (ERE) and a control DNA (ctrl). In preliminary experiments, these populations were reconstituted on the HB at a level of 2 fmoles/mm^2^ (around 150 RU). On the other side, the binding of ligand (such as E2) to the estrogen receptors induces their dimerization. This conformational change induced activation of ER and their interaction with the ERE sequence. Moreover ERα protein is known to be really difficult to store. This nuclear receptor is particularly sensitive to the denaturizing and aggregating processes [21]. Every authors working on SPR based ER/ERE interactions studies used the same ERα origin which was commercialized at 80% of purity and could be denaturized or aggregated with time [22-27]. In our study, the activated ER interacts with the DNA biosensor during injection steps and especially with the ERE sequence. This was illustrated in figure 4, where few minutes after the end of injection, 10.2 fmol/mm^2^ of activated ERα have strongly interacted with specific ERE target whereas unspecific linkage to control sequence reached 2.8 fmol/mm^2^. Whereas, due to the large variability of the control sequences in others publications, it was difficult to compare ERα/control DNA interaction responses. The level of unspecific signal (27%) obtained with our biosensor was in the range of previous publications presented results with control sequences (between 10 to 50%) [22;24;26;28].

Moreover, a particular result of our biosensor must be pointed out. (P-DNA)_2_ surface coverage has been fixed to 2 fmole/mm^2^ which corresponded to 4 fmole/mm^2^ of ERE immobilized on the lipidic matrix. When activated ER was injected at 50 nM during 15 min, the specific signal of ER/ERE interactions reached a plateau at 490 RU, i.e. around 0.49 ng/mm^2^ or 7.4 fmoles/mm^2^. In these conditions the efficiency of RE/ERE biorecognitions was up to 90%. These first results on protein/DNA interactions based on this biomolecular architecture seem to be promising and will be further investigated. Finally regeneration of RE/ERE biosensors were usually based on dsDNA stripping or denaturizing protein processes [23;25]. Our biochip offers an alternative to these methods which allow overcoming the unspecific adsorption on DNA. (P-DNA)_2_ blocks linked to ER by affinity anchorage were simply eliminated by imidazole pulses, offering a lipidic matrix available for a new DNA grafting. These preliminary results allowed us to conclude that ERα interaction is mainly ERE specific, which confers biological relevancy to our biosensor.

## 4. Experimental Section

### 4.1. Materials

LC-SPDP (Pierce Biotechnology, Rockford, USA) was used to link oligonucleotides to modified cytochrome b5 reduced by 1.4-DiThioTreitol (DTT) (Sigma Saint Louis, USA). Oligonucleotides (Eurogentec, Liege, Belgium) were able to create ERE or double strand control sequence (Ctrl) by complementary hybridizations (A1/A3 and A4/A6). P-DNA structures were purified by chromatography using DiEthylAminoEthyl (DEAE), IminoDiacetic Acid (IDA) and Sephadex G75 gels purchased from Sigma. Lipid surfaces were constituted by a mixture of DiMyristoyl-Phosphatidyl-Choline (DMPC) and 1.2-DiOleoyl-sn-Glycero-3[(N(5-amino-1carboxypenty) iminodiacetic acid)] Succinyl (DOGS) (Aventi Polar Lipids, Albaster, USA). Human recombinant Estrogen Receptor-α (ERα) (PanVera, Invitrogen Corporation, Carlsbad, USA) was conserved at -80°C into 10 μl aliquots to limit the number of freeze-thaw cycles. Estradiol-17-β (E_2_) (Sigma, St Louis, USA) was prepared at 1 μM in ethanol and stored at -20°C.

### 4.2. Supramolecular building

A1 (5′-AGTTCTTTGATCAGGTCACTGTGACCTGAACTTGCT-3′) (ε_260nm_ = 334.400 M^-1^ .cm^-1^) or A4 (5′-AGTTCTTTGATACGTCCCATCAAGTCAGACTTGCT-3′) (ε_260nm_ = 335.900 M^-1^ .cm^-1^) oligonucleotides were coupled to LC-SPDP by incubation for 17 hours in 50 mM phosphate buffer pH 7.5 (called PB) at room temperature with 1/25 molecular ratio. Excess LC-SPDP was eliminated by ion exchange chromatography (DEAE) in PB buffer. Oligonucleotides linked to the column were eluted with 1 M NaCl in PB buffer. Coupling efficiency was evaluated in reducing conditions (20 mM DTT). This reducing agent cleaves LC-SPDP, releasing thiopyridine which was quantified by spectrophotometry at 343 nm. The genetically engineered cytochrome b5 was previously described [29]. Briefly, a S24C mutation has been introduced, by directed engineering, to enable protein / linker coupling. Then, a tag of six amino acids (NGHHH) was added at the C-terminus to allow IMAC chromatography processes and grafting on DOGS.

DNA/LC-SPDP complexes were coupled to modified cytochrome b5 (ε_412nm_ = 117.000 M^-1^ .cm^-1^) through sulfhydril residue bearing by the unique cystein at position 24. This cystein was reduced by a 10-minute incubation at room temperature in DTT excess (1/10 mole/mole), which was eliminated by exclusion chromatography (Sephadex G25). DNA/LC-SPDP complexes were incubated with modified cytochrome b5 (molecular ratio 1/1) at room temperature overnight, after which DNA/LC-SPDP/b5 complexes (called P-DNA) were purified in several steps. First, complexes without cytochrome b5 were eliminated by affinity chromatography with an iminodiacetic acid column loaded using a NiCl_2_ solution (0.1 M acetate buffer pH 7.8). All cytochrome b5, bearing DNA or not, were eluted by 1 mg/ml histidine solution (PB buffer). Then P-DNA complexes were purified by ions exchange chromatography. Free cytochrome b5 were eliminated by 0.25 M NaCl (PB buffer) and P-DNA were eluted in 1 M NaCl (PB buffer). Complexes were quantified by spectrophotometric measurements at 260 and 412 nm.

A dimerization process was based on the complementary hybridization properties of DNA that lead to a structure called the (P-DNA)_2_ block. A3 (5′-AGCAAGTTCAGGTCACAGTGACCTGATCAAAGAATATATAGCAAGTTCAGGTCACAGTGACCTGATCAAAGA-3′) (ε_260nm_ = 735.700 M^-1^ .cm^-1^) or A6 (5′-AGCAAGTCGTGACTTGATGGGACGTATCAAAGAATATATAGCAAGTCGTGACTTGATGGGACGTATCAAAGA-3′) (ε_260nm_ = 739.900 M^-1^ .cm^-1^) oligonucleotides, which presented two complementary domains with respectively A1 and A4 sequences, were able to link two P-DNA complexes. Two molecular ratios between A3 or A6 and corresponding complexes were used to evaluate the most efficient dimerization process. One mole ssDNA with 3 moles of P-DNA at 4°C in PB buffer overnight. Theoretically hybridization process can lead to the building of three species: i) complexes (A1/LC-SPDP/b5)_2_-A3 or (A4/LC-SPDP/b5)_2_-A6 called respectively (P-DNA)_2_^ERE^ and (P-DNA)2^Ctrl^, ii) (A1/LC-SPDP/b5)-A3 or (A4/LC-SPDP/b5)-A6 (with only one b5) called respectively P-(DNA)_2_^ERE^ and P-(DNA)_2_^Ctrl^ and iii) A1/LC-SPDP/b5 or A4/LC-SPDP/b5 called respectively P-DNA^ERE^ and P-DNA^Ctrl^. These different species were separated by gel filtration (Sephadex G75, 0.1 M Phosphate Buffer Saline (PBS)). Spectrophotometric study was used to identify these different species by determining the characteristic A_260_/A_412_ ratio.

### 4.3. Assembling of complexes onto the chip

First, commercial gold chips SIA (GE Healthcare Life Sciences, Pittsburgh, USA) were chemically functionalized in 1 mM OM (Sigma, Saint Louis, USA) at room temperature overnight as previously published [9]. Then, 1 mM (DMPC)/(DOGS) SUVs were prepared by extrusion using a 50 nm polycarbonate membrane in PB buffer. Several DOGS/DMPC ratios were used in this study, from 0.01 to 0.1 range mole/mole, in order to modulate (P-DNA)_2_ surface density. After wetting the hydrophobic SAM with 50% ethanol, the surface was washed with 40 mM OG. Lipid vesicles spread spontaneously onto the hydrophobic surface at 25°C. Excess lipid was removed by treatment with 20 mM NaOH leading to a stable baseline. Injections of (P-DNA)_2_ onto this lipidic surface was performed at 5 μl/min.

### 4.4. REα/ DNA interaction

Interaction study was performed after 50 nM ERα dimerization in the presence of 1 nM E_2_ (4 hours incubation in PBS at 4°C). Then, receptor solution was simultaneously injected onto both (P-DNA)_2_^Ctrl^ and (P-DNA)_2_^ERE^ channels at 2 μl/min for 15 minutes.

### 4.5. SPR experiments

SPR experiments were run on BIAcore 2000 (GE Healthcare Life Sciences, Pittsburgh, USA) at 25 °C, with a flow rate of 5–50 ul/min, in PB buffer or in PBS for (P-DNA)_2_ graftings and ERα interaction experiments.

### 4.6. Spectrophotometric characterizations

Spectrophotometric study with a λ900 spectrophotometer (PerkinElmer Instrument, Waltham, USA) was performed to identify P-DNA species. For calculations, absorbance at 260 nm had to be corrected to take into account the contribution of cytochrome b5 at this wavelength (about 17% of the 413 nm absorbance value). Molar extinction coefficient at 260 nm of DNA component in (P-DNA)_2_^ERE^ complexes, for example, was $\varepsilon_{{\left(A1\right)}_{2}\;-\;A3_{260\text{nm}}}=\frac{2\times \varepsilon_{A1_{260\text{nm}}}+\varepsilon_{A3_{260\text{nm}}}}{1.67}$, so global coefficient at 260 nm for (P- DNA)_2_^ERE^ was ***ε***_(P - DNA)2ERE260nm_ = ***ε***_(A1)2 - A3 260nm_ + 2× ***ε***_b5260nm_. For P-(DNA)_2_^ERE^ molar extinction coefficient at 260 nm of DNA part was $\varepsilon_{A1-A3_{260\text{nm}}}=\frac{\varepsilon_{A3_{260\text{nm}}}}{2}+\frac{\frac{\varepsilon_{A3_{260\text{nm}}}}{2}+\varepsilon_{A1_{260\text{nm}}}}{1.67}$, so global coefficient of P-(DNA)_2_^ERE^ was ***ε**_P_*_−_ *_(DNA)_*_2_*_ERE_*_260_*_nm_* = ***ε**_A_*_1 −_ *_A_*_3_260_*nm*_ + ***ε***_b5_260_*nm*_. Molar extinction coefficient of P-DNA^ERE^ was ***ε**_P_*_−_ *_DNAERE_*_260_*_nm_* = ***ε**_A_*_1_260_*nm*_ + ***ε***_b5_260_*nm*_. The coefficient at 412 nm was only affected by number of cytochrome b5 molecules. Each species present a characteristic molar extinction coefficient ratio. Thus, all complexes could be discriminated by determining the A_260 nm_/A_412 nm_ ratio (Table 1).

### 4.7. AFM characterizations

The AFM used was a Nanoscope III (Veeco, Santa Barbara, CA). Imaging was performed in contact and oscillating contact mode (tapping^™^ mode) using NPS-oxide sharpened silicon nitride probes (Veeco) exhibiting spring constants of 0.32 N/m or 0.58 N/m at resonance frequencies of 8.5 to 9.5 kHz. For the feedback controls, typical values of set-point for imaging were between 0.5 to 1.5 V, depending on scan size and drive amplitude in oscillating contact mode. The oscillation amplitude was generally maintained at 5-10 nm away from the surface.

## 5. Conclusions

To conclude, a macromolecular assembly, called P-DNA, composed of an engineered cytochrome b5 and a modified ssDNA was synthesized and characterized. Using a complementary oligonucleotide, we have demonstrated the possibility to generate a supramolecular assembly called (P-DNA)_2_. The optimal conditions to synthesize theses blocks have been established. Theses blocks were reconstituted on a bio-functionalized gold chip and investigated in parallel with two biophysical techniques, Surface Plasmon Resonance (SPR) and Atomic Force Microscopy (AFM). From our knowledge, this is the first time that such investigations are performed in parallel on the same substrate, i.e. rough gold substrate devoted to biosensors. The construction and properties of the sensor were confirmed with both techniques, especially in term of specificity and reversibility. We guess that such approach brings sensor and nanotechnology communities in helping a better understanding of events that occur at the surface of biochip. Finally, an estrogen receptor biosensor was performed. These first results of specific biomolecular recognitions between ERE - the DNA response element -, and ER - a member of the nuclear receptor super-family - open up a new approach for developing an estrogen receptor biosensor and for drugs screening.

## Supplementary Material



## Figures and Tables

**Table 1.** **Presentation of different species of P-DNA assemblies.**The P-DNA assembling by hybridization process can lead to the building of three species: i) complexes (A1/LC-SPDP/b5)_2_-A3 or (A4/LC-SPDP/b5)_2_-A6 called respectively (P-DNA)_2_^ERE^ and (P-DNA)_2_^Ctrl^, ii) (A1/LC-SPDP/b5)-A3 or (A4/LC-SPDP/b5)-A6 (with only one b5) called respectively P-(DNA)_2_^ERE^ and P-(DNA)_2_^Ctrl^ and iii) A1/LC-SPDP/b5 or A4/LC-SPDP/b5 called respectively P-DNA^ERE^ and P-DNA^Ctrl^.

Structure	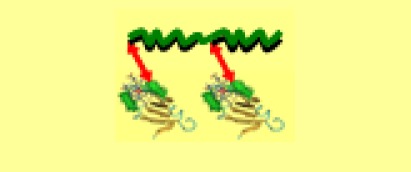	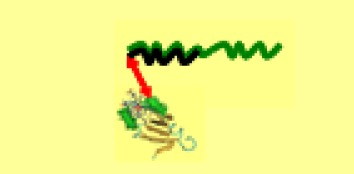	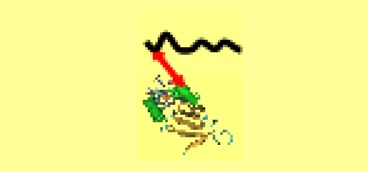
Name	(P-DNA)_2_^ERE^ or (P-DNA)_2_^Ctrl^	P-(DNA)_2_^ERE^ or P-(DNA)_2_^Ctrl^	P-DNA^ERE^ or P-DNA^Ctrl^
ERE	2	1	0
MW	75000	50000	25000
Ratio A_260_/A_412_	3.7	7	3
Legend