# Supplementary material for: Nanobioengineering and Characterization of a Novel Estrogen Receptor Biosensor
Source: Sensors (Basel). 2008 Jul 28;8(7):4413–28. doi: 10.3390/s8074413 (PMC3697183; doi:10.3390/s8074413)
Supplement: Supplementary file 1 [file sensors-08-04413-s001.pdf]

Supplementary Results 1

(P-DNA)$_2$ Supra-molecular Building

In order to determine optimal conditions to obtain the highest (P-DNA)$_2$ blocks synthesis yield, two molecular ratios of P-DNA and overlapped complementary oligonucleotides were used (2:1 or 3:1 by mole). After the hybridization and gel filtration processes, the composition of b5-DNA populations was determined by analysis of absorbance ratios (A_{260}/A_{412}) and gel electrophoresis. When a molar ratio of 2:1 was used, it leads to an incomplete synthesis of (P-DNA)$_2$ blocks. Hybridization in solution could not be fully performed under this experimental condition leading to an intermediate block called P-(DNA)$_2$. When P-DNA blocks were in excess, the equilibrium of synthesis was displaced to the synthesis of (P-DNA)$_2$. Residual P-DNA was completely removed by a chromatographic step.

After characterization of (P-DNA)2 fractions by spectrophotometry ((A_{260}/A_{412}) = 3.7) (Figure Aa), the degree of purification was evaluated by 12.5% polyacrylamide gel electrophoresis. Proteins were revealed by Coomassie blue and the DNA parts by ethidium bromide staining. In denaturing conditions (β-mercaptoethanol in sample buffer) only one 18 kDa structure corresponding to cytochrome b5 was observed (Figure Ab, lane 1). β-mercaptoethanol, a reducing agent, induced LC-SPDP/DNA releasing by the reduction of a disulfide bond between cytochrome b5 and LC-SPDP. To overcome this limitation, identification of b5-DNA complexes was performed in non-denaturing conditions (Figure Ab, lanes 2 and 3). Protein coloration of b5-DNA population revealed only the dimeric species (P-DNA)$_2^{Ctrl}$ (Figure Ab, lane 2). Ethidium bromide allowed the revelation of the double strand DNA part of this structure (Figure Ab, lane 3).

Figure A. Biochemical characterizations of (P-DNA)$_2$ blocks. **(a)** Spectrophotometric UV-visible characterization of (P-DNA)$_2$, **(b)** Electrophoresis in 12.5% polyacrylamide gel was realized in denaturing conditions (SHEtOH-β +) (lane 1), or in non-denaturating conditions (SHEtOH-β -) (lane 2 and 3). Proteins were revealed by Coomassie blue staining (lane 1 and 2). DNA parts were revealed by an UV light exposition at 312 nm after incubation in ethidium bromide (lane 3).

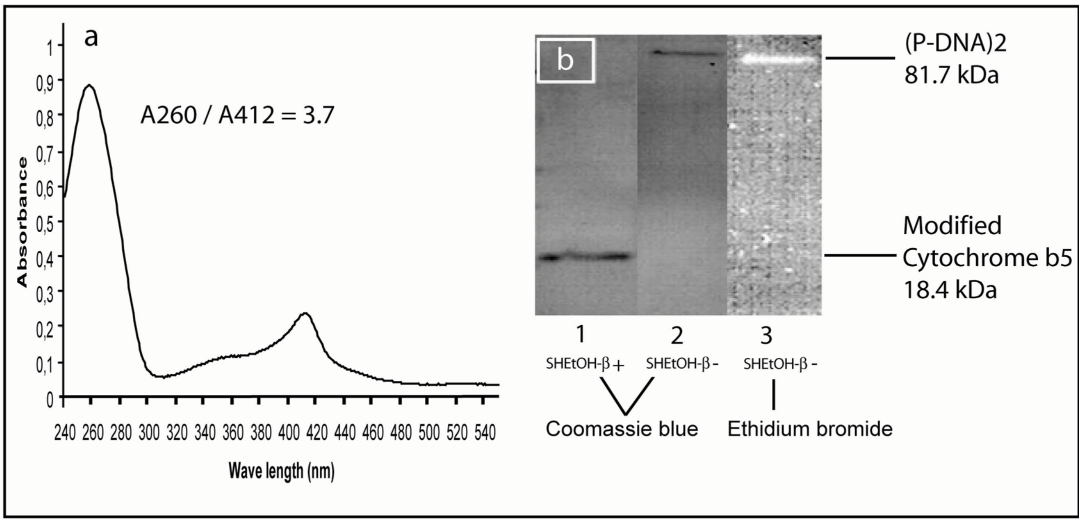

Supplementary Results 2

Interaction Mechanism Analysis of (P-DNA)$_2$ with HB

The mechanism of interaction of (P-DNA)$_2$ with HB was investigated in order to better understand the sequential steps of immobilization onto the lipidic matrix. Theoretically, (P-DNA)$_2$ entities present two histidine tags and thus, two sites of anchoring. The most convenient interaction kinetic model must take into account this particularity. We have faced to face association/dissociation results with "Langmuir 1:1" and "Bivalent analyte" models (Figure B). Optimal fitting was obtained using the Bivalent analysis model for all studies involving (P-DNA)$_2$ blocks. This model, proposed in *BiaEvaluation* 3.05 software, corresponds to the following mechanism of interaction:

phase 1: A + B ↔ AB

phase 2: AB + B ↔ AB2

with A corresponding to the analyte "(P-DNA)$_2$" and B to the ligand "lipid anchor DOGS". Thus, results of fitting are consistent with the theory that (P-DNA)$_2$ initially binds to one lipid anchor (DOGS) in the lipid matrix through one of its histidine tags. This preliminary interaction with the lipid matrix leads to a free histidine tag that can react with a second lipid anchor. When DOGS were in excess in the lipid matrix, such coupling was optimized giving higher affinities than those observed for monomeric P-DNA.

Figure B. Kinetic analysis of (P-DNA)$_2$ grafting onto the surface. Superimposition of kinetic models and experimental (P-DNA)$_2$ data. Experimental data (grey curve) and resulting fits with the Langmuir 1:1 model (blue curve) and the bivalent model (purple curve) purposed in *BiaEvaluation* 3.05.

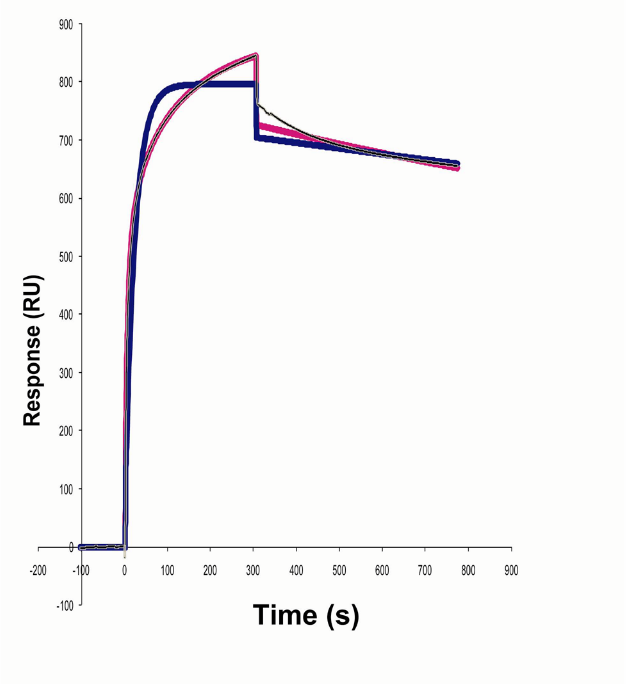

Supplementary results 3

AFM Characterization of Hybrid Bilayer

Figure C. Establishment of supported lipid layer onto the OM modified gold surface. **(a)** AFM images in contact mode of commercial gold chip surface presenting spherical particles of gold (diameters of 30 to 50 nm) **(b)** The profile section along the white line (on the image a). **(c)** Certain heterogeneity were visualized after 1h lipid vesicles fusion onto the hydrophobic gold surface but disappeared after a short treatment with 20 mM NaOH, **(d)** z scale corresponds to 8 (a) and 100 (b,c) nm.

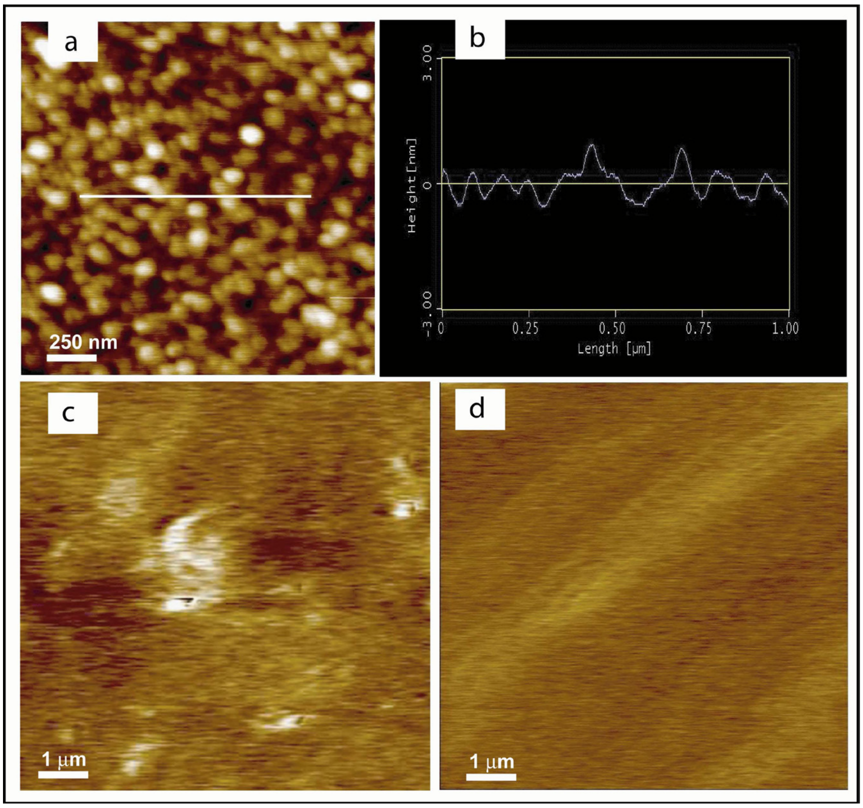

Supplementary Method 1

Gel Electrophoresis

Purification degree was evaluated by 12.5% polyacrylamide SDS-gel electrophoresis. Classical denaturing SDS-PAGE (Sodium Dodecyl Sulfate-PolyAcrylamide Gel Electrophoresis) was performed on 2 µg of b5-Ctrl sample. Proteins were heated to 95 °C for 5 minutes in sample buffer (0.2% SDS, 0.001% bromophenol blue, 62.5 mM Tris-HCl, 10% glycerol and 10 mM β-mercaptoethanol (SHEtOH-β). A non-denaturing electrophoresis was also performed on 2 µg of proteins by not heating and using β-mercaptoethanol free sample buffer. Gels were incubated in Coomassie blue solution overnight and destained for two hours in 10% acetic acid, 30% methanol solution. Next, a bath in 0.5 µg/mL ethidium bromide was performed overnight, and DNA presence was revealed by a UV light exposition at 312 nm.
